# Medical Opinion and Movement

**Published:** 1910-12-31

**Authors:** 


### Medical Opinion and Movement.

### Immunity against Malta Fever.

At a meeting of the Société de Biologie Drs. Vincent and Collignon made a report upon their experimental work with the object of immunising goats against infection with the micrococcus melitensis. Three-days-old cultures were emulsified with saline and ether. The mixture was then left for twenty-four hours at the temperature of the laboratory. The ether was evaporated at 38° C., and the culture killed in this way was used as the vaccine. In two animals three consecutive injections were made subcutaneously, and induced a moderate elevation of temperature, the first injection producing the most marked reaction. In two other goats one injection only was made intravenously. Only a slight febrile reaction resulted in these animals. In order to determine whether these injections had conferred immunity upon the animals, intravenous injections were made of 4 cubic centimetres of a living and very active culture of the micrococcus melitensis. The first two goats that received three injections did not develop any abnormal symptoms, and they have since developed well and are in perfect health. Their serum agglutinates well. The other two goats that were given only one intravenous injection showed much less resistance to infection.

### Dysphagia in Tuberculous Laryngitis.

Dr. R. Hoffmann first proposed about two years ago to treat the painful symptoms of tuberculous laryngitis by anæsthetising the internal branch of the superior laryngeal nerve by means of alcoholic injections. Dr. G. Roth, of Reichenhall, now reports upon the treatment of thirty-three cases in the *Münchener Medizinische Wochenschrift*. The chief indication for the injections is the distressing dysphagia that accompanies this condition. In such cases a pathognomic painful spot is found between the hyoid bone and the crycoid cartilage corresponding to where the sensory branch of the superior laryngeal nerve perforates the thyro-hyoid membrane. After disinfection with alcohol a somewhat blunt needle is pushed through the skin at this point for a depth of 1½ cm., and is then directed upwards and outwards. When the nerve is reached the patients complain of pain radiating towards the ear; 1 to 2 cm. of 85-per-cent. alcohol, raised to 45° C., are injected. In the cases of Dr. Roth the condition had already existed for two or three months, in spite of the usual methods of treatment. Swallowing liquids or only the saliva was more painful than swallowing solid or semi-solid food. In all but one case the injections produced a beneficial effect upon the dysphagia. In some the effect was immediate, but in the majority of cases relief was only obtained after one or two days. Many, who had not been able previously to take any nourishment for several days, could after the injection swallow food and even fluids without pain. The effect of the injection lasted generally about a week—at the most three weeks. In one case the symptoms disappeared altogether after one injection. In all the other cases the symptoms returned, but in a less degree, and the injections must be repeated. After each injection the effect is more rapid and persists longer. The author does not consider that fever or extreme weakness are contra-indications against the injections.

### Post-operative Cancer Recurrences.

There is still no satisfactory explanation of the fact that recurrences of cancer *in situ* may take place even after the most extensive excision of the neoplasm. Reasons put forward are that there is a microscopic extension of the cancer beyond the limits of the growth, or that the neighbouring lymphatic glands have become infected, or that the operative wound is inoculated by the knife. Dr. Pierre Nodal, however, puts forward another hypothesis which is based upon observations and microscopic preparations from a case of this nature. An account of his work appears in the *Bulletine et Memoires de la Société Anatomicale de Paris*. The case was an epithelioma at the base of the neck, which recurred five times after five operations, the last being followed by fulguration. The means of contamination put forward by the author, as demonstrated by his preparations, consist in the disgorgement into the operative wound of the lymph contained in the adjoining lymphatic spaces and holding in suspension cancerous cells. The hypothesis suggested is that the lymphatics undergo a sort of massage in the course of the operation which results in contaminating the lymph with the most peripheral cancerous elements, which are also the youngest and most active. This contamination may take place in spite of the most extensive removal of the whole suspected area, and in spite of all precautions against direct infection nothing can prevent this lymph from flowing back towards the wound after its closure. This process can be seen best in the acute forms of breast cancer, from which a large number of preparations have been made by Dr. Nodal to demonstrate it. Some of them show not only free cancer cells in the lymphatics, but also in the blood capillaries, and especially in those surrounding the lymphatic glands. The author is of opinion that these intracapillary cancer cells arrive there also *viâ* the lymphatic channels. If these facts and theories be correct they will form an added terror to the operative surgeon, and the question must needs present itself how such massage of the lymphatics can best be avoided during operation.

### Inflammation and Malignant Disease.

A very scholarly address on this subject, delivered by Mr. C. B. Lockwood before the Manchester Medical Society, is printed in the *Medical Chronicle*. In it the author maintains three propositions: First, that inflammation precedes cancer, sarcoma, and endothelioma; next, that inflammation causes these growths; and last, that inflammation and malignant disease owe their origin to the same causes. He defines inflammation as an increase in the number of cells in a part caused by an irritant. An irritant is further defined as that which, being applied to living tissues, causes their cells to multiply. It is true that this reads like argument—or at least definition—in a circle; but Mr. Lockwood manages to make it sound quite plausible. Of all irritants, the most frequent and universal are the bacterial irritants; but other irritants, electrical, mechanical, and so on, may also set up prolonged inflammation and be followed by cancer. The tongue, the scrotum, the breast, *x*-ray dermatitis, and other familiar clinical sites of malignant disease furnish texts for the thesis that inflammation precedes malignant growth. The relations of chronic cholecystitis to cancer of the gall bladder, and of chronic gastric ulcer to carcinoma of the stomach, are also quoted in the same way. Numerous cases are also mentioned in which sarcoma of the testis has supervened upon long-continued inflammation due to syphilis and other causes; and two cases of endothelioma of the glands of the neck following chronic inflammation of undetermined nature have come under the author's observation. Mr. Lockwood may justly claim to have shown that inflammation precedes (in many cases) malignant growth; but he is very far indeed from having proved the second and third of his three propositions.

### The Pathology of Dropsy.

IN a very long paper on "The Blood and Urine in Dropsy," published in the *Medical Chronicle*, Dr. R. Lakin deals with many aspects of his complicated theme. His general conclusions on œdema are worth attention, especially as they contain a point in treatment which has, it appears, already been suggested by Dr. Melland, but is as yet little known. With œdema, says Dr. Lakin, NaCl is practically always retained in the body; as œdema disappears the excess of salt is removed, and its output rises above the intake. That this NaCl retention is, however, not necessarily dependent upon the presence of œdema, or *vice versa*, is shown by certain facts. Thus when dropsy has disappeared the NaCl excretion is not uncommonly markedly subnormal, and also there may be marked œdema without any retention of NaCl. Also in certain cases the output of NaCl has been observed to increase as the dropsy becomes more marked. The conclusion is that salt excretion has not always the same significance in the genesis of œdema. Imperviousness of the kidneys to chlorides and consequent retention of the latter is the cause of many cases of œdema; but many other cases are due to entirely different causes, and even where chloride retention has some effect it may be playing but a subsidiary part. Hence the varying opinions as to the efficacy of salt-free diet in cases of dropsy. Now NaCl output is reduced by 50 per cent. in many cases of simple chlorosis, even without œdema, and Dr. Melland has had beneficial results in this disease from a salt-free diet. Hence Dr. Lakin is led to the hypothesis that primary salt retention, in association with other factors, may play an important part in the ætiology sychlorosis.

### Absorption of Sugar in Nutrient Enemata.

THE value of sugar as a diffusible and nourishing food for rectal administration has long been firmly believed in by many physicians. In the *Deutsche Archiv. fur Klin. Med.*, von Halasz gives the details of experiments designed to estimate the relative merits of different sugars used for this purpose. He injected them into the colon by the ordinary funnel and soft tube, and analysed the urine and the subsequent fæcal evacuations. Solutions of strengths up to 40 per cent. were used, and the volume of each enema was 500 c.c. Dextrose is retained better than cane sugar, and lævulose is inferior to both in this respect. Lactose and maltose are not well absorbed, especially the latter, which is not well borne by the colon; the former is as well retained as dextrose. Cane sugar and dextrose are absorbed readily, and to an extent greater than is generally supposed. Thus in one case 144 gm. of dextrose were absorbed from a single enema of 40 per cent. strength, and cane sugar is almost equally readily taken up. Of the latter, strengths of 10 to 30 per cent. answer best. In no case did the urine contain sugar after these experiments. Since it may be alleged that the disappearance of such large quantities of sugar introduced into the rectum may be caused by their destructive fermentation, the author mentions experiments which show that this accounts for but little of the lost carbohydrate; and other observations on dogs are quoted to show that after the small intestine has been ligatured and divided off from the large, sugar solutions are absorbed from the colon just as readily as before. That some four to five ounces of such an excellent tissue-sparing food as dextrose can be taken up from a nutrient enema is an addition to our knowledge worth having. In the *Zentralblatt f. Chirurgie* Berendes extols the same sugar for subcutaneous and intravenous feeding. The maximum which can thus be introduced without setting up glycosuria is about 50 gm. per day. In this way 300 calories may be taken up; caution is necessary, as glycosuria ensues if the treatment is continued for several consecutive days.

### "Therapeutic Neoplasms."

DRS. REYNIER and Masson have recently drawn the attention of the Paris Academy of Medicine to the unsuitability of the usual compresses and dressings employed in hospital practice and generally. Repeated sterilisation causes them to become fluffy, with the result that a few strands of the material are apt to become detached from the main mass and so to adhere to the tissues surrounding the dressings. Much irritation is then set up, so much so that in three cases observed by them an inflammatory tumour was formed which led at first to the diagnosis of a recurrence or metastasis. In each of the three cases cited an operation had been performed for the removal of a suspected malignant tumour. A few days later a second tumour appeared in the neighbourhood of the operation lesion. The rapidity with which this second growth appeared led them to doubt of its being a true recurrence, and microscopical examination revealed the presence of cotton threads, obviously derived from the dressings used at the operation. Round these foreign bodies there had been set up an intense tissue reaction, thereby leading to the formation of a "therapeutic neoplasm." The remedy for the evil is in the hands of the manufacturer of dressings. The authors recommend the hemming of compresses on the ground that although this may add to the already heavy cost of dressings the increased expenditure will be the lesser of two evils.

### An Unconscious Pregnancy and Labour.

Dr. Fierera Cabrera has published in the *Rivista de Med. y. Cir. practicas* the following interesting case. The patient, a woman of twenty-three, married for three years and suckling a child born eleven months after marriage, was suddenly taken ill in the middle of the night with pain in the lower abdomen of a colicky nature, which no treatment could relieve. A doctor called in a few hours later found the abdomen hard and containing a tumour of fairly large proportions. Thinking that the colicky pains were due to indigestion he ordered enemata, massage, soothing applications, and finally, as none of these had much effect, a draught containing opium and potassium bromide. Even this last proved of no avail in checking the pains, which only increased in severity, until finally, after a strong expulsive effort, the well-formed head of a child appeared at the vulva. Mother and father alike could hardly believe their senses, so unexpected was the result. The mother declared that but for the fact that she saw the child she could not have believed that it was hers, for she never had any suspicion that she was pregnant. Leaving on one side the comical aspect of this case, the author attempts to explain how it was that the mother was unaware of her condition. It is, of course, true that her amenorrhœa did not strike her as extraordinary in view of the fact that she was nursing her first child; and again, being naturally inclined to corpulency, she put down her increase in abdominal girth to an aggravation of her tendency. The movements of the fœtus she must have mistaken for flatulence. A number of cases of this kind have been reported by various observers, including Desgranges, Duquesnel, Fodéré Vogel, Klein, and Brouardel, in which no pathological cause could be found to account for this condition of ignorance. It would thus seem an established fact that a woman may be pregnant without knowing it, and may enter into labour when thinking she is the subject of severe colic—facts which, from a medico-legal point of view, are of great interest.

### Chronic Catarrh of Nasal Passages.

A recent number of the *New York Medical Journal* contains an article by Dr. Beverley Robinson, of New York, with reference to the treatment of chronic nasal catarrh. He believes that the main causes of the condition are impaired general nutrition, bad hygienic surroundings, nasal obstruction, and follicular disease of the naso-pharynx. Contributory causes are to be found in dust, winds, exposure, draughts, improper clothing, excessive eating, drinking and smoking. The first step in treatment should therefore be to remedy impaired nutrition and insanitary surroundings. The form of nasal obstruction most commonly found is that due to hypertrophied turbinates, with or without septal deviation. The septum must, in a number of cases, be straightened or removed before a cure can be effected; while it is almost always necessary to remove the thickened mucous membrane covering the turbinated bones, which is best done by repeated cauterisation with some mild acid. It is wrong to use strong acids, as they lead to the formation of scar-tissue and scabs, as well as to impairment of the olfactory sense in some cases. After the nasal passages have been cleared sufficiently, the use of a mild alkaline cleansing spray daily for some weeks is good treatment. Follicular disease of the naso-pharynx is best combated by the use of a solution of one part of tincture of iodine to two of glycerine, applied on alternate days for two or three weeks, and later at less frequent intervals. If there be adenoids of any size present they must be removed, as well as enlarged tonsils.

### Cammidge's Reaction.

Cammidge's pancreatic reaction in the urine has been investigated recently from a new aspect by Drs. Whipple, Chaffee, and Fisher in the Johns Hopkins Hospital laboratories. They have produced experimental pancreatitis, acute and chronic, and other lesions in animals, and have then applied the reaction to the urine of those cases in which pancreatic lesions had resulted and those in which there were no such lesions in the pancreas. Their conclusions are as follows: (1) The Cammidge test is of little value in establishing a diagnosis of acute pancreatitis in dogs. If the test is negative, it is pretty strong evidence against an acute pancreatitis. (2) The Cammidge test is of even less value in the condition of chronic pancreatitis in dogs, and may be consistently absent even in extreme grades of this disease. (3) A positive Cammidge test is not infrequent in normal dogs and men. (4) The Cammidge test is almost constantly present in chloroform poisoning in dogs—a condition in which there is extensive liver necrosis and cell autolysis. (5) The Cammidge test may be present in cases of pneumonia or in any condition where there is active cell-destruction and autolysis. (6) The Cammidge test may be produced experimentally almost at will. (7) The melting-point of the crystals varies under different conditions, indicating that the substance or substances are not constant. (8) The method is open to various errors, and too much depends upon the personal equation, particularly in the interpretation of the various crystals. (9) The method is time consuming, and the results do not seem to warrant the expenditure of the time and materials.

